# Effects of Hydrocolloid Type and Mixing Temperature on the Properties of Pea-Based Texturized Vegetable Protein Burgers

**DOI:** 10.3390/foods15213785

**Published:** 2026-10-22

**Authors:** Andrea Bresciani, Francesca Girotto, Alessandra Marti

**Affiliations:** 1 Department of Food, Environmental and Nutritional Sciences (DeFENS), Università Degli Studi di Milano, Via G. Celoria 2, 20133 Milan, Italy; 2 Department of Environmental Science and Policy (ESP), Università Degli Studi di Milano, Via G. Celoria 2, 20133 Milan, Italy; francesca.girotto.89@hotmail.it

**Keywords:** meat analogues, texturized vegetable protein, pea proteins, hydrocolloids, psyllium, texture

## Abstract

Given the growing interest in alternatives to cellulose-derived hydrocolloids, this study evaluated the effects of psyllium compared with methylcellulose (MC), hydroxypropyl methylcellulose (HPMC), and their combination on the functional and rheological properties of pea-based texturized vegetable protein (TVP) and the technological properties of the corresponding plant-based burgers. The influence of mixing temperature (5–25 °C) was also investigated. Hydrocolloid addition had limited effects on water solubility and oil absorption, whereas psyllium addition resulted in lower water absorption capacity, and the hydrocolloid mixture increased foaming capacity and stability of the tested pea-based TVP system. Viscosity was significantly affected by temperature and hydrocolloid type, while torque depended mainly on hydrocolloid type and its interaction with temperature. Before cooking, burger hardness was primarily influenced by mixing temperature, whereas after cooking, it was mainly affected by hydrocolloid type. Psyllium-containing burgers showed comparatively lower cooked hardness at 10 and 15 °C, while MC- and HPMC-containing burgers showed higher cooked hardness. Cooking loss was influenced by temperature, hydrocolloid type, and their interaction, with MC showing marked temperature-dependent behaviour and the hydrocolloid mixture providing the lowest losses at 15 °C. Overall, hydrocolloid functionality depended on hydration conditions, mixing temperature, and subsequent thermal treatment, and no formulation showed consistently superior performance across all technological properties.

## 1. Introduction

The growing global population, projected to reach nearly 10 billion by 2050, alongside increasing environmental concerns, is driving a substantial shift in dietary patterns and food production systems [1]. The livestock industry, while central to traditional protein supply chains, is resource-intensive, contributing significantly to greenhouse gas emissions, land use, and water consumption [1]. These challenges underline the urgent need to identify and develop sustainable alternative protein sources to reduce dependence on conventional meat production [2]. Among the most promising developments are meat analogues, products designed to replicate the sensory, nutritional, and structural characteristics of conventional meat using non-animal proteins [3]. Yet, most plant proteins are globular and require texturization processes to align and reorganize their conformation in a way that better replicates the look and mouthfeel of meat [4,5]. Among different processing technologies, extrusion is the most mature and studied technique [6] for transforming plant proteins into fibrous, meat-like structures suitable for use in meat analogues. Specifically, low-moisture (<40%) extrusion results in expanded products known as texturized vegetable protein (TVP), which are dry, porous matrices that require rehydration before use [7]. Among their various applications in the development of meat analogues, such as burgers, nuggets, and sausages [8], burgers represent the most widespread one [3]. However, TVP alone often cannot guarantee sufficient cohesion, water retention, and thermal stability during processing and cooking of meat analogues [9,10]. To address these limitations, hydrocolloids are commonly employed as stabilizers, thickening agents, and emulsifiers, enhancing the texture and rheological behaviour of plant-based formulations [11]. These polysaccharides help reinforce the structural matrix by interacting with plant proteins, promoting network formation, and improving the overall cohesiveness of the system [12,13]. One of the most widely used hydrocolloids in food applications is methyl cellulose (MC), a chemically modified cellulose derivative [9]. MC is soluble in cold water, allowing for effective binding during mixing, and undergoes gelation upon heating, thereby stabilizing the structure of the product during cooking [14,15]. This thermal gelation contributes not only to shape retention but also to moisture retention [14,16], both of which are critical for replicating the sensory qualities of meat. An alternative is hydroxypropyl methyl cellulose (HPMC), another thermo-responsive modified cellulose. Differences in substitution pattern and molecular characteristics between MC and HPMC result in distinct hydration, aggregation, and thermal gelation behaviours [17]. HPMC typically has a higher molecular weight, which enables it to form more extensive and stronger gel networks. Moreover, HPMC exhibits a higher thermal gelation threshold (typically between 75 and 90 °C) compared with MC (40–50 °C), indicating greater thermal stability [17,18].

As an alternative to cellulose-derived hydrocolloids, attention has also turned to psyllium, a natural, soluble dietary fibre with excellent water-binding and viscosity-enhancing properties [19]. Unlike synthetic polymers, whose gelation often follows simpler, well-defined transitions, the gelation of food polymers such as psyllium involves multiple overlapping phenomena, including coil-helix conformational transitions, hydrogen bonding, and physical entanglement [20]. Psyllium can develop weak gel behaviour upon hydration through physical interactions and chain entanglement, differing from the thermally induced gelation characteristic of MC and HPMC [20,21,22].

Processing temperature is another critical parameter that influences both the functionality of hydrocolloids and protein structuring in meat analogues [23,24]. During formulation and cooking, temperature regulates protein hydration, unfolding, aggregation, and the development of both non-covalent (e.g., hydrogen bonding, hydrophobic interactions, electrostatic forces) and covalent disulphide bonds [15]. Recent evidence suggests that decreasing the processing temperature can enhance the cohesiveness of protein-rich legume-based gels [15], whereas during cooking, hydrocolloids may assist as physical cross-linkers that bind protein filaments [11].

Previous studies have explored the role of different hydrocolloids (such as guar gum, k- and iota-carrageenan, xanthan gum, low acyl gellan gum, hydroxypropyl starch, sodium alginate, carboxymethyl cellulose, low methylated pectin, cross-linked tapioca starch, and locust bean gum) in soy protein isolate systems [12], as well as in wheat- and pea-based protein isolates [11], highlighting their potential in structuring plant-based matrices. However, the use of psyllium in combination with texturized pea protein remains largely unexplored. Although psyllium has been investigated in pea-based sausages [22], the effects of psyllium on the technological properties of pea-TVP burgers under different processing conditions remain poorly characterized. In particular, limited information is available on how mixing temperature interacts with hydrocolloid type to determine the properties of the raw matrix and its subsequent behavior during cooking.

Based on the current knowledge gap in plant-based burger formulation, this study aimed to investigate the role of hydrocolloids (i.e., psyllium, MC, and HPMC) in pea-based TVP systems and burgers and to assess how mixing temperature influenced their functional, rheological, cooking, and textural properties. Particular attention was given to the interaction between hydrocolloid type and mixing temperature and to whether psyllium could provide functionality comparable to that of MC and HPMC under the investigated conditions.

## 2. Materials and Methods

### 2.1. Materials

Texturized vegetable protein (TVP) derived from yellow pea (*Pisum sativum* L.) in granular form (70% protein; 9% moisture) was kindly provided by Giusto Faravelli S.p.A. (Milan, Italy). The hydrocolloids employed in this study were methylcellulose (MC, E461; Tylopur MCE-100TS), hydroxypropyl methylcellulose (HPMC, E464; SFE 4000), and psyllium (*Plantago* spp., Psyllium Pure White 99%–100 mesh; 88% dietary fiber); they were kindly provided by GB Studio (Basaluzzo, Alessandria, Italy).

For functional and mixing properties, the TVP was ground for 30 s using a laboratory grinder (M20 Universal Mill, IKA, Staufen, Germany) to obtain a powder with a particle size of 750 µm. For the burger preparation, the TVP was ground for 30 s using a mini chopper (Desire Mini Chopper, Russell Hobbs, Manchester, UK), and only the fraction with particle size greater than 4000 µm was retained.

The ground TVP was mixed with each hydrocolloid individually (99:1 *w*/*w*), corresponding to 1% (*w*/*w*) hydrocolloid in the dry mixture, or with a ternary blend of the three hydrocolloids in equal proportions (1:1:1), corresponding to approximately 0.33% (*w*/*w*) of each hydrocolloid in the dry mixture. The hydrocolloid level was intentionally kept relatively low (1% *w*/*w* of the dry TVP–hydrocolloid mixture) to assess whether differences in hydrocolloid functionality could be detected at low inclusion levels under otherwise standardized formulation conditions. The 1:1:1 ratio was selected to provide a balanced contribution of the three hydrocolloids within the fixed total concentration.

To ensure homogeneous distribution, mixing was carried out using a professional planetary mixer (Kenwood Cooking Chef XL, Havant, UK) operated at low speed for 2 min. The TVP alone was used as a control.

### 2.2. Methods

#### 2.2.1. Functional Properties

Water absorption capacity (WAC) and oil absorption capacity (OAC) were determined according to Bresciani et al. [25]. Briefly, 3 g of sample was mixed with 30 mL of distilled water for WAC determination or 30 mL of sunflower oil for OAC determination. The suspensions were vortex-mixed for 30 s on three occasions, with 10 min intervals between mixing steps. Samples were subsequently centrifuged at 2500× *g* for 30 min at 22 °C (NEYA 8 centrifuge; REMI Elektrotechnik Limited, Mumbai, India). The supernatant was removed, and the solid fraction was recovered. WAC and OAC were expressed as g water/g sample and g oil/g sample, respectively.

The water solubility index (WSI) was determined according to Yu et al. [26] using the aqueous supernatant obtained during WAC determination. The aqueous supernatant was transferred to pre-weighed crucibles, pre-evaporated on a heating plate for 10 min, and subsequently dried overnight at 105 °C. After drying, the crucibles were weighed to determine the number of soluble solids. The WSI was expressed as g/100 g.

Foaming capacity (FC) and foaming stability (FS) were determined according to Yadav et al. [27]. Briefly, 0.5 g of sample was dispersed in 25 mL of distilled water in graduated cylinders. The cylinders were sealed with paraffin film and manually shaken continuously for 2 min. They were then placed on a flat surface, and the foam volume was recorded after 5 min for determination of FC and again after 60 min for determination of FS. FC and FS were expressed as percentages.

Three independent TVP–hydrocolloid mixtures were prepared for each formulation and analysed separately; therefore, n = 3 represents three independent experimental replicates.

#### 2.2.2. Rheological Properties

Viscosity measurements were carried out using a ViscoQuick instrument (Brabender GmbH & Co. KG, Duisburg, Germany). Each sample (20 g) was dispersed in 100 mL of distilled water, with both components scaled to a 14% TVP moisture basis. Samples consisted of TVP and water, with or without the addition of 1% hydrocolloid. Measurements were conducted under controlled stirring (250 rpm). Each test started at 30 °C, and the temperature was decreased to the target temperature (5, 10, 15, or 25 °C) within 5 min, corresponding to cooling rates of −5, −4, −3, and −1 °C/min, respectively. The target temperature was then maintained for the remaining 15 min, for a total test duration of 20 min. Viscosity (expressed in Brabender Units, BU) was monitored throughout the test and the value recorded at the 10th minute, selected as a standardized comparison point after 5 min of equilibration at the target temperature.

Mixing properties were assessed using a Farinograph-E (Brabender GmbH & Co. KG, Duisburg, Germany) equipped with a 50 g mixing bowl. Before each measurement, the mixing bowl was equilibrated at the selected temperature (5, 10, 15, or 25 °C) for 20 min. In each test, 30 g of TVP (alone or with 1% hydrocolloid) was combined with 60 g of distilled water previously equilibrated at the target temperature. The reported temperature therefore refers to the water temperature and bowl temperature. Samples were mixed at different temperatures (5 °C, 10 °C, 15 °C, or 25 °C) at 63 rpm for 20 min, and torque (expressed in BU) was continuously recorded. Torque was recorded at the 10th minute of mixing, when the curve had reached a stable plateau, and this value was therefore selected for comparison among samples.

Thermo-mechanical behaviour was studied by using a Mixolab device (ChopinTechnologies, Villeneuve-la-Garenne, France). For the assay, 35 g of TVP (either alone or supplemented with 1% hydrocolloid) was tested at 100% constant hydration, with the amount of distilled water adjusted to account for the initial moisture content of the sample. The settings used in the test were as follows: (1) the temperature was kept constant at 30 °C for 8 min; (2) it was increased to 90 °C at a rate of 4 °C/min and held for 7 min; (3) it was then reduced to 50 °C at the same rate and held for an additional 5 min. The mixing speed during the entire analysis was 80 rpm. Torque (N·m) was recorded continuously throughout the analysis.

All tests were performed in triplicate.

#### 2.2.3. Burger Preparation and Characterization

Before burger preparation, all ingredients were conditioned in a climate chamber (Memmert GmbH, Schwabach, Germany) until the designated pre-conditioning temperature (5 °C, 10 °C, 15 °C, or 25 °C) was reached. Once the temperature was stabilized, 200 g of distilled water was weighed, and 1 g of hydrocolloid was dispersed into it under continuous stirring. Subsequently, 99 g of TVP (or 100 g in the formulation without hydrocolloids) was added to the hydrocolloid solution. The actual temperature of the mixture was not measured after mixing; therefore, the temperatures reported in this study refer to the pre-conditioning temperature of the ingredients.

The resulting mixture was then kneaded at speed 6 for 5 min using a planetary mixer (Cooking Chef XL, Kenwood, Havant, UK) equipped with a K-beater attachment. After 2.5 min, mixing was interrupted and a manual homogenization step was performed by redistributing material from the sides and bottom of the bowl, after which mixing was resumed for the remaining 2.5 min. The same procedure was applied to all samples.

Immediately after mixing, 120 g portions were formed into burgers using a hamburger press (Life Srls, Naples, Italy; diameter 9.7 cm), resulting in burgers with a height of 1.8 ± 0.04 cm.

The oven (SelfCookingCenter, Rational International AG, Landsberg am Lech, Germany) was preheated to 180 °C before cooking. Burgers were placed, immediately after preparation, in the center of a perforated tray and cooked for 8 min; the core temperature at the end of cooking was approximately 75 °C. Core temperature was measured using a temperature probe, inserted into the geometric centre of the burger. Burgers were turned once after 4 min during cooking.

For each formulation × temperature condition, two independent 300 g bulk mixtures were prepared. Two burgers were obtained from each bulk mixture, resulting in two independent experimental replicates, each represented by two burger subsamples.

Burgers were analyzed both before and after cooking to determine moisture content, water activity, and hardness.

Moisture content was determined using a moisture analyzer (MA 210.R, Radwag Wagi Elektroniczne, Radom, Poland). Approximately 3 g of sample was analyzed at 130 °C until the sample weight changed by less than 1 mg over 10 s. Measurements were performed in triplicate.

Water activity (aw) was measured using a LabMaster-aw instrument (Novasina AG, Lachen, Switzerland) at 25 °C. Approximately 2 g of sample was placed in the instrument sample cup, and aw was recorded after equilibrium had been reached according to the instrument stability criterion.

Texture analysis was conducted using a texture analyzer (TA.XT Plus, Stable Micro Systems, Godalming, UK) fitted with an HDP/BSK shear blade, and maximum force (hardness) was recorded at 60% strain. Cooking losses were calculated based on the difference between the pre- and post-cooking weights [28].

Cooking loss was calculated from burger weight before and after cooking according to the following equation:

Cooking loss (%) = [(W_raw − W_cooked)/W_raw] × 100

where W_raw and W_cooked are the burger weights before and after cooking, respectively.

Measurements were performed on the individual burgers. For statistical analysis, values obtained from burgers originating from the same independently prepared bulk mixture were averaged, and the bulk preparation was considered the experimental unit (n = 2 independent preparations per formulation × temperature condition).

#### 2.2.4. Statistical Analysis

All data were expressed as means ± standard deviation. Statistical analysis was performed using Statgraphics Plus 5.1 software. Functional properties were analysed by one-way analysis of variance (ANOVA), with formulation as the fixed factor, followed by Tukey’s HSD test for multiple comparisons (*p* ≤ 0.05).

Viscosity, mixing torque, uncooked and cooked burger hardness, uncooked and cooked moisture content, uncooked and cooked water activity, and cooking loss were analysed using two-way fixed-effects ANOVA, with formulation and mixing temperature as fixed factors. Formulation comprised five levels (TVP without hydrocolloids, TVP + psyllium, TVP + MC, TVP + HPMC, and TVP + mix), whereas mixing temperature comprised four levels (5, 10, 15, and 25 °C). The full factorial model included the main effects of formulation and mixing temperature and their interaction (formulation × mixing temperature). Uncooked and cooked measurements were treated as separate response variables.

Separate one-way ANOVAs were also used to compare formulations within each mixing temperature and mixing temperatures within each formulation. When a significant formulation × mixing-temperature interaction was detected, interpretation focused on these simple effects, which were evaluated by one-way ANOVA followed by Tukey’s HSD test. Within each response variable, the experimental design was balanced, with the same number of replicates for each formulation × mixing-temperature combination.

Functional and rheological measurements were performed in triplicate (n = 3). For burger characterization, hardness, moisture content, water activity, and cooking loss were measured on all burgers. Normality of model residuals was assessed using the Shapiro–Wilk test, and homogeneity of variances was evaluated using Levene’s test. Statistical significance was established at *p* ≤ 0.05.

Principal component analysis (PCA) was performed using JMP 5.0 software on standardized variables (zero mean and unit variance) to account for differences in measurement units and scales. The variables included viscosity, mixing torque, cooking loss, uncooked burger hardness, and cooked burger hardness, with uncooked and cooked hardness treated as separate variables. The data matrix consisted of the mean values obtained for each formulation × mixing-temperature combination, with each combination representing one observation in the PCA. Thus, five formulations evaluated at four mixing temperatures resulted in 20 observations. PCA was used to explore multivariate relationships among variables and the distribution of samples according to formulation and mixing temperature.

## 3. Results

### 3.1. Functional Properties

The effects of hydrocolloid addition on the functional properties of pea-based TVP are reported in Table 1.

TVP showed a WAC of 2.3 g/g, a WSI of 1.14 g/100 g, an OAC of 2.1 g/g, a foaming capacity (FC) of 27.8%, and a foaming stability (FS) of 27.0%.

The WAC of TVP falls within the range previously reported for seven types of texturized pea protein (1.5–2.9 g/g; [7]). By contrast, its OAC was approximately twice the value reported by Hong et al. [7], being 2.1 g/g compared with 0.82–1.04 g/g. These results indicate a comparatively greater fat-binding capacity of the TVP used in the present study. In addition to cultivar-related differences, protein isolation procedures and texturization conditions may substantially affect the functional properties of TVP [29,30,31,32].

Although hydrocolloid addition is generally expected to increase water absorption because of the high hydrophilicity of these polymers [11], the inclusion of 1% MC or HPMC did not significantly affect WAC. Similarly, no significant variation was observed when MC and HPMC were used as part of the hydrocolloid mixture. Conversely, the formulation containing psyllium showed a significantly lower WAC than the TVP.

Hydrocolloid addition did not significantly affect the WSI or OAC, suggesting that neither the amount of water-soluble material released from the TVP nor its fat-binding capacity was substantially modified under the conditions investigated. The individual addition of MC, HPMC, or psyllium also produced only limited effects on foaming properties. However, the formulation containing the hydrocolloid mixture exhibited the highest FC and FS values. In particular, FC was significantly higher than in the formulations containing MC or psyllium, whereas FS was significantly higher than in the untreated TVP and in the samples containing MC or psyllium.

### 3.2. Rheological Properties

The impact of hydrocolloids and temperature on the viscosity of TVP is reported in Figure 1. The addition of MC and HPMC generally resulted in higher viscosity values than TVP, whereas psyllium showed a less consistent response. At 10 °C, the TVP + psyllium formulation exhibited significantly lower viscosity than the TVP + MC and TVP + HPMC samples, while no significant differences among hydrocolloid-containing formulations were detected at the other temperatures. Across all formulations, viscosity increased as temperature decreased from 25 to 5 °C. The largest numerical variation was observed between 10 and 15 °C. The TVP + mix formulation showed the smallest numerical variation over the investigated temperature range, although its temperature response did not differ statistically from that of the other formulations because the interaction term was not significant. Two-way ANOVA revealed significant main effects of both temperature (*p* < 0.0001) and hydrocolloid type (*p* = 0.0002) on viscosity, whereas the temperature × hydrocolloid interaction was not significant (*p* = 0.2233). Overall, viscosity increased as temperature decreased from 25 to 5 °C, and formulation also influenced viscosity. Although individual pairwise differences among formulations were observed at some temperatures, the non-significant interaction indicates that the effect of formulation did not significantly differ across the investigated temperatures.

Regarding the effect of hydrocolloids and temperature on mixing properties (Figure 2), the TVP + MC formulation showed the lowest torque at 5 °C and the highest value at 10 °C. At 15 °C, the TVP + mix formulation exhibited a significantly higher torque than TVP and TVP + MC but did not differ from TVP + HPMC or TVP + psyllium. At 25 °C, TVP + mix showed the highest torque, TVP the lowest value, and the formulations containing individual hydrocolloids showed intermediate values. Torque did not vary significantly across temperatures for the HPMC- and psyllium-containing systems. The response of TVP + MC between 5 and 10 °C may reflect temperature-dependent changes in MC hydration, solubilization, water distribution and matrix consistency; however, these mechanisms were not directly assessed. The comparatively high torque of the TVP + mix formulation at 15 and 25 °C suggests that the simultaneous presence of MC, HPMC and psyllium increased resistance to mixing at the higher temperatures investigated.

Two-way ANOVA showed a significant effect of hydrocolloid type (*p* < 0.0001) and a significant temperature × hydrocolloid interaction (*p* < 0.0001) on torque, whereas the main effect of temperature was not significant (*p* = 0.0711).

No significant differences among formulations were observed for the Mixolab parameters (Figure S1). The torque-time profiles obtained during heating and cooling were therefore comparable among the TVP and the hydrocolloid-containing formulations.

### 3.3. Burger Characterization

Plant-based burgers were characterized for textural properties before (Figure 3a) and after (Figure 3b) cooking, as well as for cooking loss (Figure 4).

For uncooked burger hardness, two-way ANOVA revealed a significant main effect of mixing temperature (*p* = 0.0005), whereas hydrocolloid type and the temperature × hydrocolloid interaction were not significant (*p* = 0.4031 and *p* = 0.0588, respectively). This indicates that the hardness of the uncooked burgers was mainly influenced by mixing temperature rather than by hydrocolloid formulation. Some significant differences among formulations were detected at individual temperatures. At 5 °C, TVP + MC showed lower (although not significant) hardness than the other formulations, whereas at 10 °C the TVP alone and TVP + psyllium exhibited the lowest values and TVP + mix the highest. However, no significant differences among formulations were observed at 15 or 25 °C. Overall, psyllium did not produce a consistent effect on uncooked hardness relative to the other hydrocolloids across the investigated temperatures.

After cooking, the statistical response was reversed. Hydrocolloid type significantly affected burger hardness (*p* = 0.0001), whereas mixing temperature and the temperature × hydrocolloid interaction were not significant (*p* = 0.9131 and *p* = 0.0759, respectively). Cooked hardness therefore remained substantially unchanged across the investigated mixing temperatures, while differences were mainly associated with formulation. Pairwise comparisons within individual temperatures identified lower hardness for psyllium than for MC and HPMC at 10 and 15 °C; however, these differences should not be interpreted as a temperature-dependent psyllium effect because the overall interaction was not significant. No significant differences among formulations were detected at 5 or 25 °C.

Cooking losses were significantly affected by temperature (*p* < 0.0001), hydrocolloid type (*p* = 0.0492), and their interaction (*p* < 0.0001). No significant differences among formulations were observed at 5 °C. MC was associated with the lowest and highest cooking losses at 10 °C and 25 °C, respectively. The mixture of the hydrocolloids was effective in decreasing the cooking loss both at 15 °C and 25 °C. Overall, psyllium provided a comparatively temperature-stable response but did not consistently reduce cooking losses relative to MC, HPMC, or the hydrocolloid mixture.

Moisture content and water activity of uncooked and cooked burgers are reported in Table S1. No significant differences among formulations were observed for either parameter under the conditions investigated.

### 3.4. Principal Component Analysis

Principal component analysis provided an integrated overview of the relationships among rheological properties and burger performance (Figure 5). The first two principal components accounted for 61% of the total variance, with PC1 and PC2 explaining 35.6% and 25.4%, respectively. Cooking loss and raw burger hardness were closely aligned on the positive side of PC1, indicating a strong positive association between these variables. Torque was oriented in the opposite direction, indicating a negative association with raw hardness and cooking loss within the experimental dataset. Cooked hardness loaded mainly on the positive side of PC2 and in the opposite direction to raw hardness and cooking loss, confirming that pre-cooking consistency was not directly related to the texture developed after thermal processing. Viscosity also contributed positively to PC2, although its association with cooked hardness was only partial.

In the score plot, untreated TVP samples were mainly positioned on the positive side of PC1 and were therefore more closely associated with raw hardness and cooking loss. The mixed-hydrocolloid formulation was predominantly located on the negative side of PC1, closer to the torque vector, indicating a greater association with mixing resistance. Psyllium-containing samples showed a similar tendency but were more widely dispersed, suggesting a stronger dependence on processing conditions. MC samples were mainly associated with positive PC2 scores and cooked hardness, whereas HPMC samples were positioned closer to the centre of the plot and showed a more intermediate profile. Nevertheless, the partial overlap among formulations indicates that hydrocolloid type alone did not fully explain sample differentiation, and that mixing temperature also contributed to the observed variability.

## 4. Discussion

This study investigated how hydrocolloid type and mixing temperature affected the functional properties of pea-based TVP systems and the technological characteristics of the corresponding plant-based burgers.

Pea-derived TVP was selected as the protein matrix, reflecting the growing interest in pea proteins as versatile and potentially sustainable ingredients for plant-based foods [33].

Hydrocolloids were intentionally evaluated at a relatively low inclusion level to investigate their functionality under low-dose conditions. Consequently, the magnitude of the observed effects should be interpreted within the concentration range tested, as hydrocolloid functionality is concentration-dependent [34].

Functional properties were evaluated as an initial broad characterization of the TVP–hydrocolloid systems, covering hydration, lipid-binding, solubility, and foaming behaviour, rather than as direct predictors of burger performance. Burger technological performance was evaluated separately through cooking loss and instrumental texture measurements. WAC is relevant to water retention and may influence juiciness and mouthfeel, whereas OAC reflects the ability of the protein matrix to retain lipids, with potential effects on flavour retention and texture [35,36]. The WSI provides an indication of the amount of soluble material released from the matrix and may indirectly reflect the extent of structural disruption or protein solubilization [37].

Foaming capacity and stability were included as complementary indicators of the techno-functional behaviour of the TVP–hydrocolloid systems, as they can reflect changes in interfacial properties and continuous-phase viscosity [38]. However, because the burger formulation and processing conditions were not specifically designed to generate an aerated structure and air incorporation was not quantified, these properties should not be considered direct predictors of burger cooking loss and hardness.

Hydrocolloids may nevertheless modify foaming behaviour indirectly by modifying water distribution and phase viscosity, as well as binding and gel formation during processing [11].

Under the conditions used in this study, hydrocolloid addition mainly affected WAC and foaming properties, whereas the WSI and OAC remained unchanged. The absence of significant changes in the WSI indicates that hydrocolloids did not substantially alter the amount of soluble material released from the TVP under the conditions of the assay. Similarly, the unchanged OAC suggests that the affinity of the matrix for the lipid phase was not significantly modified by the tested binders.

Although hydrocolloids are generally expected to increase water retention because of their hydrophilic nature [11], neither MC nor HPMC significantly modified WAC at the concentration tested (1%). However, the hydrocolloid level used in the present study was relatively low, and hydrocolloid functionality is concentration-dependent. Therefore, the absence or magnitude of specific effects observed here should be interpreted within the concentration investigated. By contrast, the formulation containing psyllium showed a lower WAC. This result is not intuitive, given the high intrinsic affinity of psyllium for water [19,39]. One possible explanation is that the rapid hydration and marked thickening ability of psyllium altered water distribution within the suspension. Water may have remained preferentially associated with a psyllium-rich continuous phase rather than being retained within the porous TVP structure after centrifugation. However, this hypothesis should be confirmed through dedicated studies on water distribution and microstructure. Steric effects or reduced accessibility of water to hydrophilic sites within the protein matrix may also have contributed [40]. These mechanisms remain hypothetical, however, because interactions between psyllium and texturized pea protein have been scarcely investigated.

As regards the foaming properties, the combination of the three hydrocolloids produced the highest foaming capacity and stability. This might suggest that the three polymers together promoted bubble formation and retention, possibly by increasing continuous-phase viscosity (Figure 1) and decreasing liquid drainage from the foam lamellae, in agreement with previous observations on mixed-hydrocolloid systems [41]. However, these results should be regarded as complementary information on the functional behaviour of the TVP–hydrocolloid system rather than as evidence of improved burger performance. Because air incorporation was not quantified in the burgers, no direct relationship between foaming properties and burger texture or cooking behaviour can be established. Accordingly, FC and FS should be considered complementary descriptors of the TVP–hydrocolloid systems rather than direct indicators of burger performance.

The rheological tests provided complementary information on the behaviour of the TVP systems under different hydration regimes. Most studies on plant proteins and hydrocolloid-containing matrices focus on heating because of the relevance of protein denaturation, aggregation, and gelation [42,43]. Comparatively less attention has been paid to temperatures below ambient conditions, despite their technological relevance during mixing, forming, handling, and refrigerated storage of plant-based burgers [44]. Specifically, the temperature range of 5–25 °C was selected to represent pre-cooking processing conditions ranging from chilled processing (5 °C) to ambient handling (25 °C), while 10 and 15 °C were included as intermediate conditions to assess temperature-dependent changes during hydration and mixing.

Under excess-water conditions (Figure 1), viscosity was significantly affected by both temperature and hydrocolloid type, whereas their interaction was not significant. This response cannot be attributed exclusively to reinforcement of the protein network [45], as temperature-dependent changes in continuous-phase viscosity, molecular mobility, and protein-polysaccharide interactions may all contribute [40,46]. Under excess water, the rheological response may be governed mainly by matrix hydration capacity and polymer-solvent interactions. The higher viscosity measured at low temperatures may be relevant to processing behavior, as plant-based burger formulations are commonly mixed, formed and handled under chilled conditions, and greater viscosity may contribute to consistency and shape retention before cooking [47]. However, these technological effects were not directly evaluated in the present study.

The contrasting behaviour observed under excess-water and limited-hydration conditions likely reflects the different extent to which polymer hydration and competition for water can develop. Under excess-water conditions, the contribution of the hydrocolloids may be governed primarily by hydration and continuous-phase viscosity, whereas under limited hydration, competition for water between TVP and hydrocolloids may become increasingly important and affect matrix consistency and resistance to mixing [46,48].

A different response emerged under limited-hydration conditions (Figure 2), where restricted water availability may have increased competition for water between proteins and hydrocolloids. Under these conditions, farinograph torque reflected the overall resistance of the matrix to mixing. This resistance may depend on water distribution, polymer hydration, protein-polysaccharide interactions, and resistance to shear [46,49]. Hydrocolloid swelling may further increase the consistency of the concentrated matrix, although its contribution depends on hydrocolloid type and formulation conditions [49]. In this environment, hydrocolloid hydration and swelling can modify water distribution, matrix consistency, and resistance to mixing. The significant temperature × hydrocolloid interaction indicates that the influence of temperature depends on formulation. Unlike viscosity, torque did not show a uniform response to temperature across all formulations. The marked response of MC between 5 and 10 °C may reflect temperature-dependent changes in MC hydration, solubilization, water distribution, and matrix consistency. The comparatively high torque of the mixed-hydrocolloid formulation at 15 and 25 °C suggests that the simultaneous presence of MC, HPMC, and psyllium increased resistance to mixing under these conditions.

The Mixolab test (Figure S1) was used to assess the thermomechanical response of the system during heating and cooling and to determine whether hydrocolloid addition altered its resistance to thermomechanical treatment. The absence of significant differences in the parameters indicates that the effects detected under excess-water and limited-hydration conditions did not translate into measurable changes in the thermomechanical response during heating and cooling. However, the lack of significant differences may also reflect limited sensitivity of the Mixolab conditions to differences in matrix consistency detected during low-temperature mixing. Further studies are needed to assess whether modified Mixolab testing conditions could better discriminate among the formulations. It should be noted that the functional and rheological measurements were performed on TVP ground to approximately 750 µm, whereas a much coarser fraction (>4000 µm) was used for burger preparation. Since particle size can affect hydration, solubility, exposed surface area, and protein–hydrocolloid interactions, these measurements should be regarded as complementary descriptors of the TVP–hydrocolloid systems rather than as direct predictors of burger behaviour.

The properties of the plant-based burgers showed different responses to mixing temperature and hydrocolloid formulation before and after cooking, reflecting different underlying mechanisms. The absence of significant differences in moisture content and water activity (Table S1) suggests that differences in overall water content were unlikely to be the primary explanation for the observed variations in hardness (Figure 3) and cooking losses (Figure 4). These variations may instead reflect differences in water distribution, water-matrix interactions, and/or protein–hydrocolloid organization, although these aspects were not directly assessed.

Before cooking, burger hardness was primarily affected by mixing temperature, while the overall effect of hydrocolloid type was not significant. The significant effect of mixing temperature on uncooked hardness may reflect temperature-dependent differences in hydration kinetics, molecular mobility, and continuous-phase viscosity. Moreover, the formulation-specific differences observed at some individual temperatures were not sufficiently consistent to produce a significant overall hydrocolloid effect. Thus, before cooking, the observed differences in matrix consistency may have been associated with temperature-dependent hydration and swelling phenomena occurring during mixing.

Upon cooking, the observed changes in texture may have reflected protein aggregation, water redistribution, and hydrocolloid-specific thermal responses, although these mechanisms were not directly assessed. Accordingly, the influence of mixing temperature disappeared, whereas hydrocolloid type became the main factor affecting hardness. The absence of a significant mixing-temperature effect after cooking suggests that thermal treatment may have reduced the differences associated with the initial mixing conditions. Protein aggregation, water redistribution, and hydrocolloid-specific thermal behaviour may have contributed to the development of cooked texture. Statistically, cooked hardness was primarily associated with formulation.

The significant main effect of formulation on cooked hardness indicates that binder type influenced the texture developed after cooking, whereas the non-significant formulation × temperature interaction provides no evidence that this effect depended on mixing temperature. However, the lower cooked hardness of the psyllium-containing burgers, particularly in samples mixed at 10 and 15 °C, indicates that psyllium produced a less firm cooked matrix than MC or HPMC. This may be related to differences in thermally induced association and gelation, gel rigidity, water retention and compatibility with the TVP structure. A lower instrumental hardness does not necessarily indicate poorer structuring, as it may also correspond to a softer and more deformable texture that could be desirable in a plant-based burger [47,50,51]. Conversely, the higher hardness associated with MC and HPMC may reflect greater resistance to deformation resulting from thermal association, gel formation, or reduced mobility of the aqueous phase.

The comparison between raw and cooked hardness highlights that, before cooking, texture was mainly controlled by the physical conditions of mixing and hydration. After cooking, the specific functionality of the hydrocolloid became more important. This distinction may be technologically relevant because hardness of the uncooked sample is relevant to forming, handling, and shape retention, whereas hardness after cooking provides an instrumental measure of the resistance of the cooked matrix to deformation.

Cooking losses, which, in the present formulation, were expected to consist predominantly of water and soluble solids, since no lipid was added, were affected by temperature, hydrocolloid type and their interaction, indicating that the effectiveness of each binder depended on the temperature applied during mixing. In particular, MC reduced cooking losses at 10 °C but produced the highest value at 25 °C, whereas the hydrocolloid mixture was most effective at 15 °C. The marked temperature dependence of MC may be related to changes in polymer hydration, solubilization and compatibility with the TVP matrix before cooking [52]. At 10 °C, MC may have promoted a water distribution and matrix consistency that limited the release of water and soluble material during heating. At 25 °C, however, greater hydration or redistribution of MC within the continuous phase may have interfered with the organization of the protein-rich matrix or increased the amount of loosely retained water, resulting in greater cooking losses. The comparatively low cooking losses of the mixed-hydrocolloid formulation at 15 °C may reflect complementary effects of MC, HPMC and psyllium on water binding, phase viscosity and matrix cohesion. Psyllium can hydrate and swell at low temperatures [21], whereas cellulose derivatives contribute to thickening before heating and undergo further association during thermal treatment [14,15]. Their combined presence may therefore have promoted a more favourable distribution of water before cooking. At 25 °C, however, the mixture was significantly more effective mainly in comparison with MC and did not show a general reduction in cooking loss compared with all the other formulations. These mechanistic interpretations should be regarded as hypotheses, since water distribution within the burger matrix and the structural organization of the protein–hydrocolloid network were not directly assessed in the present study.

The PCA provided an integrated view of the relationships among rheological properties and burger performance. The close alignment of cooking loss and raw hardness suggests that conditions associated with a firmer uncooked matrix also tended to show greater losses during cooking, whereas the opposite orientation of torque indicates that higher resistance to mixing was associated with lower raw hardness and cooking loss. Cooked hardness was separated from raw hardness, indicating that, within the present dataset, uncooked burger hardness was not closely associated with the texture developed after thermal treatment. The partial overlap among formulations further indicates that neither hydrocolloid type nor mixing temperature alone was sufficient to explain sample differentiation. Rather, the overall technological response resulted from specific formulation-temperature combinations. These multivariate results support the conclusion that no single functional or rheological parameter can predict burger performance. Moreover, within the formulation and processing conditions investigated, psyllium did not consistently reproduce the effects observed with MC or HPMC across all measured technological properties.

Overall, psyllium showed effects comparable to those of cellulose derivatives for some responses, but not consistently across WAC, viscosity, torque, cooking loss, and cooked hardness. Its potential as an alternative hydrocolloid therefore appears to depend on the specific technological property considered and should be further evaluated across different formulations and inclusion levels.

## 5. Conclusions

The results showed that the technological effects of hydrocolloids in pea-based TVP systems depended on both the property considered and the processing conditions. The comparison between uncooked and cooked burgers indicated that different factors governed product behaviour before and after thermal treatment. Mixing temperature mainly influenced pre-cooking consistency, whereas cooked texture was primarily associated with hydrocolloid type, although the underlying mechanisms were not directly investigated.

No single functional or rheological parameter was sufficient to predict burger performance, suggesting that the observed technological response reflected the combined effects of formulation, mixing conditions, and thermal treatment. The relative importance of formulation and mixing temperature depended on the response considered. Mixing temperature was the main factor associated with uncooked burger hardness, whereas formulation primarily influenced cooked hardness. By contrast, the significant formulation × temperature interactions observed for mixing torque and cooking loss indicate that, for these responses, formulation and mixing temperature should be considered jointly.

Under the formulation and processing conditions investigated in this study, psyllium affected several technological properties of the TVP systems and burgers, but its response was not consistently similar to that of MC or HPMC across the measured outcomes. These conclusions are specific to the formulation and hydrocolloid level investigated; further studies across a broader concentration range are needed to establish concentration-dependent functionality. Moreover, further studies should assess the sensory properties and consumer acceptability of the most promising hydrocolloid–temperature combinations after their incorporation into complete burger formulations.

## Figures and Tables

**Figure 1:**
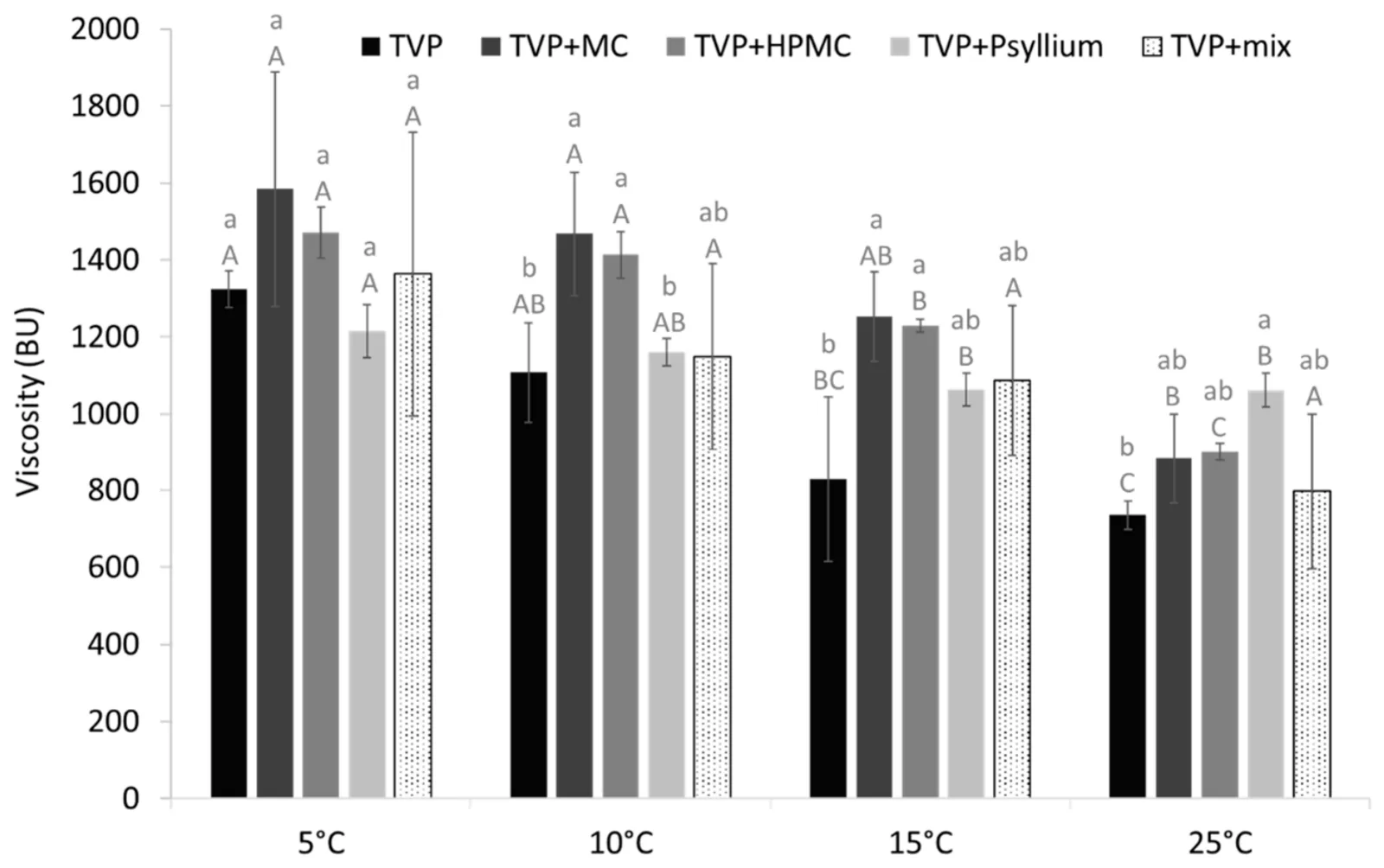
Viscosity, expressed as Brabender Units (BU), measured at the 10th min in pea-based texturized vegetable protein (TVP) systems without hydrocolloids or supplemented with 1% (*w*/*w*) methylcellulose (MC), hydroxypropyl methylcellulose (HPMC), psyllium, or their ternary mixture (mix; MC:HPMC:psyllium = 1:1:1), at 5, 10, 15, and 25 °C. Values are expressed as means ± standard deviation (n = 3). Different lowercase letters indicate significant differences among formulations at the same temperature, whereas different uppercase letters indicate significant differences among temperatures within the same formulation (Tukey’s HSD test, *p* ≤ 0.05).

**Figure 2:**
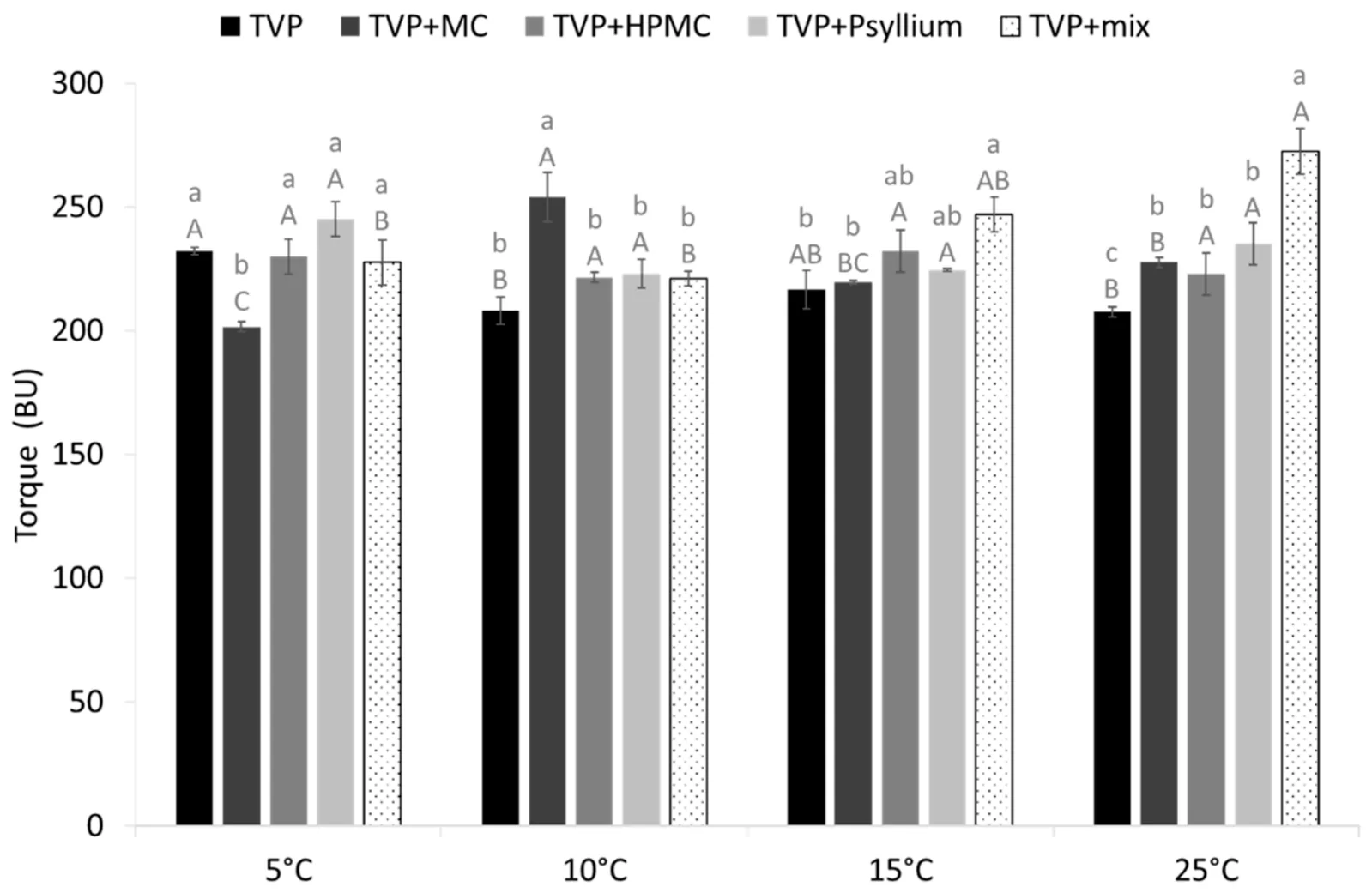
Mixing torque, expressed as Brabender Units (BU), measured at the 10th min in pea-based texturized vegetable protein (TVP) systems without hydrocolloids or supplemented with 1% (*w*/*w*) methylcellulose (MC), hydroxypropyl methylcellulose (HPMC), psyllium, or their ternary mixture (mix; MC:HPMC:psyllium = 1:1:1), at 5, 10, 15, and 25 °C. Values are expressed as means ± standard deviation (n = 3). Different lowercase letters indicate significant differences among formulations at the same temperature, whereas different uppercase letters indicate significant differences among temperatures within the same formulation (Tukey’s HSD test, *p* ≤ 0.05).

**Figure 3:**
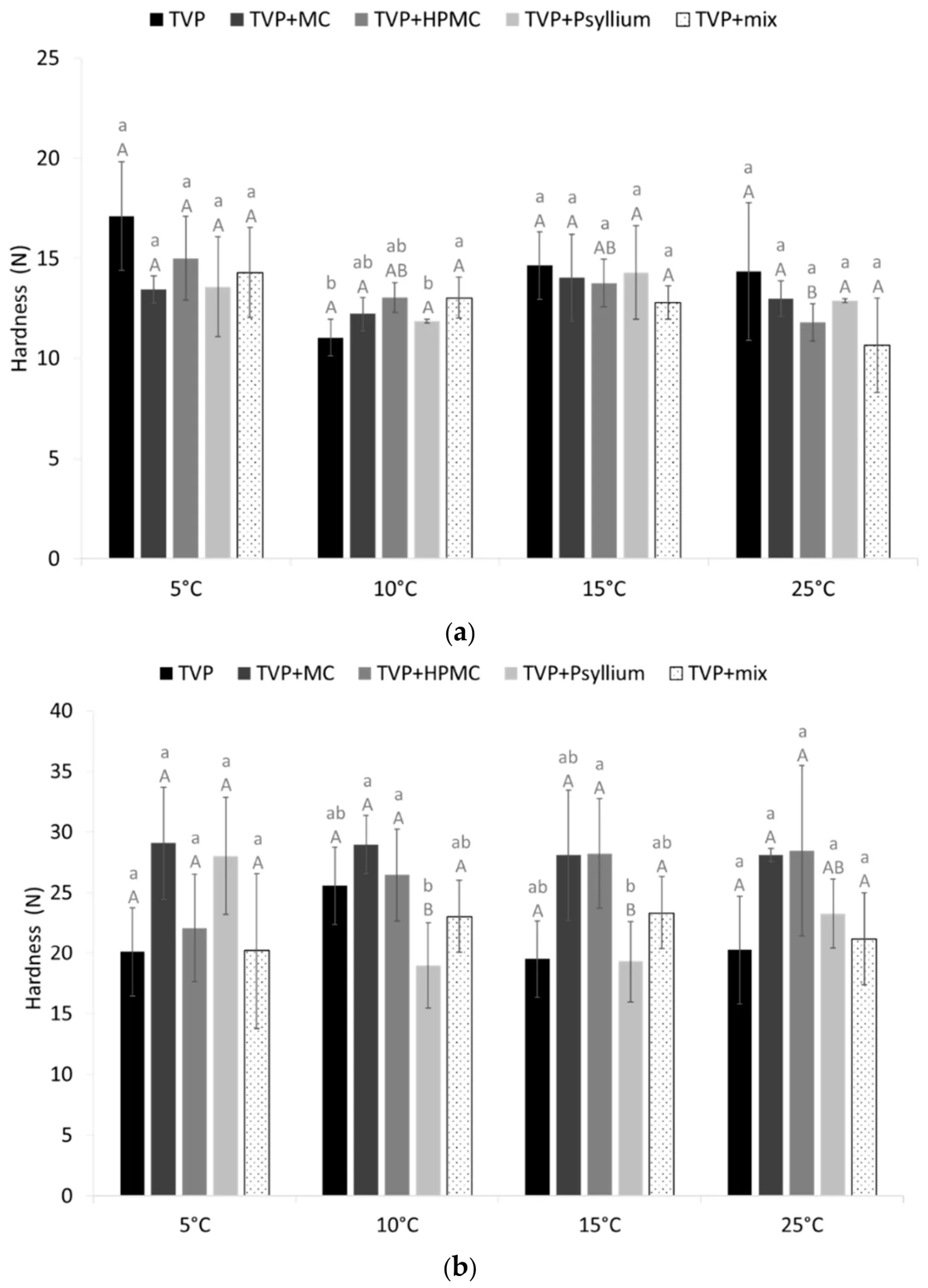
Hardness (N) of uncooked (**a**) and cooked (**b**) pea-based texturized vegetable protein (TVP) burgers prepared without hydrocolloids or with 1% (*w*/*w*) methylcellulose (MC), hydroxypropyl methylcellulose (HPMC), psyllium, or their ternary mixture (mix; MC:HPMC:psyllium = 1:1:1), after mixing at 5, 10, 15, and 25 °C. Values are expressed as means ± standard deviation of two independent bulk preparations (n = 2); two burger subsamples were analysed per preparation. Different lowercase letters indicate significant differences among formulations at the same mixing temperature, whereas different uppercase letters indicate significant differences among mixing temperatures within the same formulation (Tukey’s HSD test, *p* ≤ 0.05).

**Figure 4:**
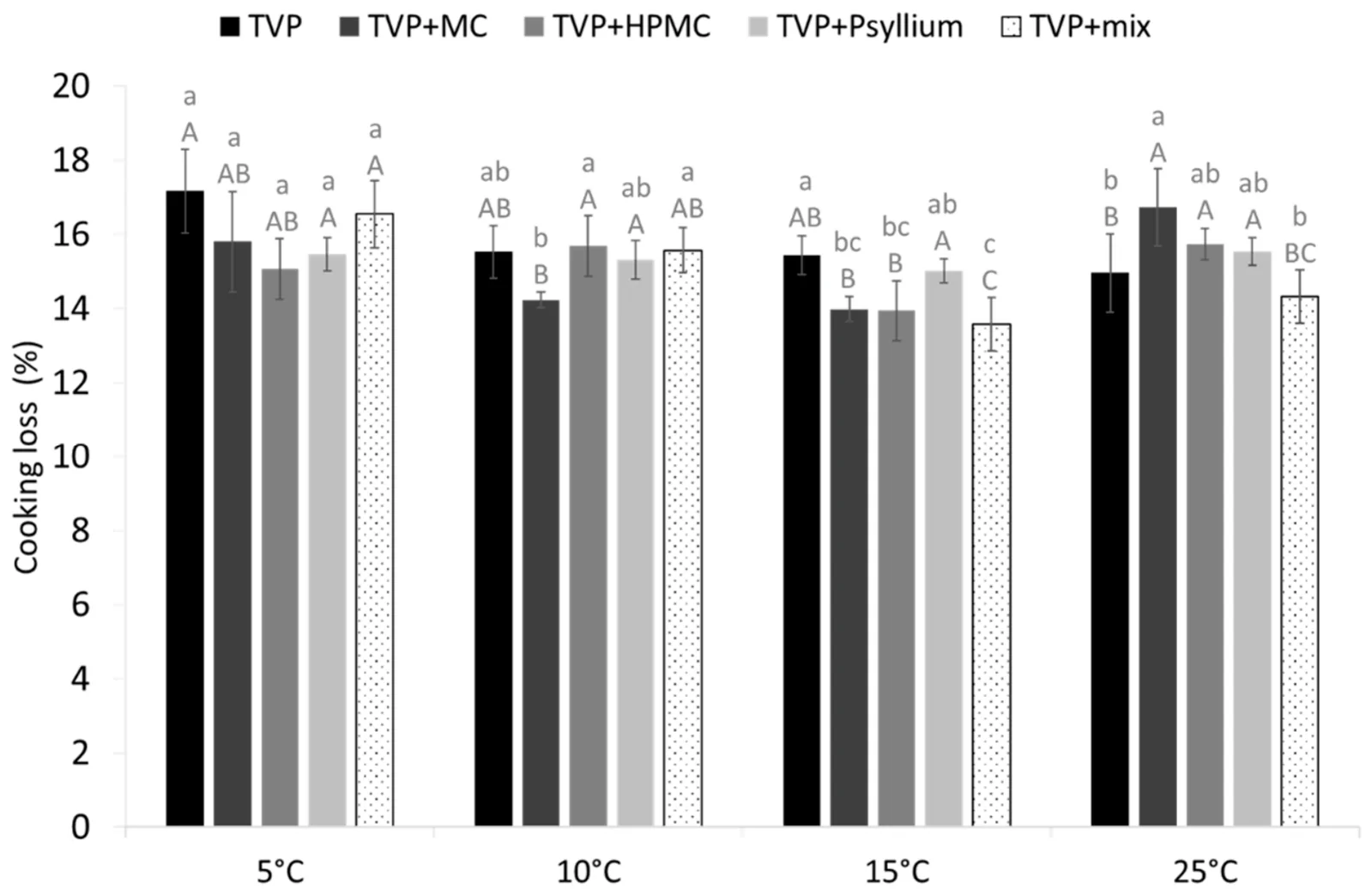
Cooking loss (%) of pea-based texturized vegetable protein (TVP) burgers prepared without hydrocolloids or with 1% (*w*/*w*) methylcellulose (MC), hydroxypropyl methylcellulose (HPMC), psyllium, or their ternary mixture (mix; MC:HPMC:psyllium = 1:1:1), after mixing at 5, 10, 15, and 25 °C. Values are expressed as means ± standard deviation of two independent bulk preparations (n = 2); two burger subsamples were analysed per preparation. Different lowercase letters indicate significant differences among formulations at the same mixing temperature, whereas different uppercase letters indicate significant differences among mixing temperatures within the same formulation (Tukey’s HSD test, *p* ≤ 0.05).

**Figure 5:**
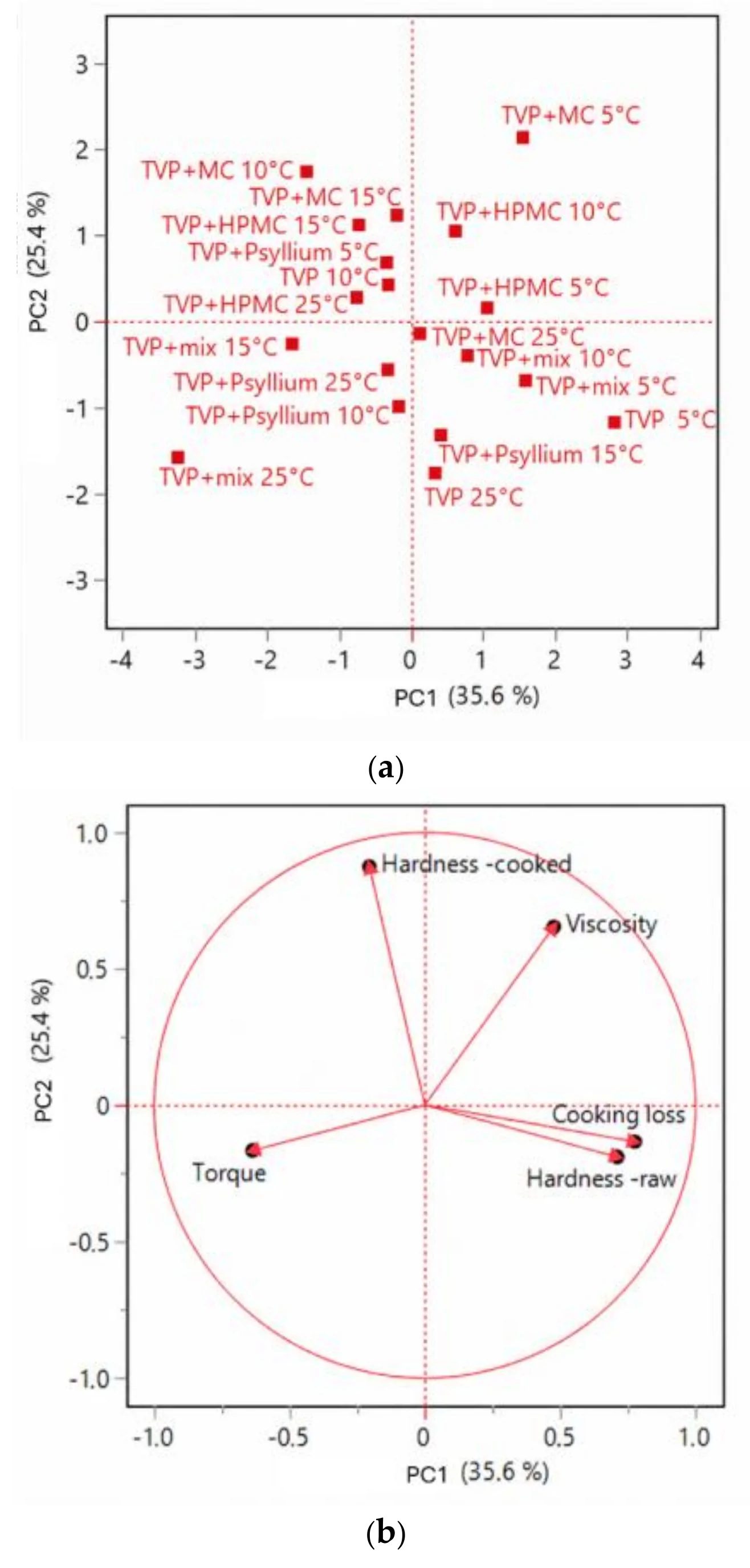
Principal component analysis (PCA) score plot (**a**) and loading plot (**b**) based on standardized mean values of viscosity, mixing torque, cooking loss, uncooked burger hardness, and cooked burger hardness. Variables were standardized to zero mean and unit variance prior to PCA. Each observation in the score plot represents one hydrocolloid formulation × mixing-temperature combination. TVP, texturized vegetable protein; MC, methylcellulose; HPMC, hydroxypropyl methylcellulose; mix, ternary mixture of MC, HPMC, and psyllium (1:1:1). PC1 and PC2 accounted for 35.6% and 25.4% of the total variance, respectively.

**Table 1:** Functional properties of texturized pea protein (TVP) without hydrocolloids and supplemented with 1% (w/w) methylcellulose (MC), hydroxypropyl methylcellulose (HPMC), psyllium, or their ternary mixture (mix; MC:HPMC:psyllium = 1:1:1, corresponding to approximately 0.33% of each hydrocolloid).

Sample	WAC (g/g)	WSI (g/100 g)	OAC (g/g)	FC (%)	FS (%)
**TVP**	2.3 ± 0.02 b	1.14 ± 0.01 a	2.1 ± 0.06 a	27.8 ± 0.35 ab	27.0 ± 0.01 a
**TVP + MC**	2.3 ± 0.10 b	1.27 ± 0.10 a	1.9 ± 0.08 a	26.5 ± 0.71 a	26.8 ± 0.35 a
**TVP + HPMC**	2.4 ± 0.04 b	1.17 ± 0.01 a	1.9 ± 0.04 a	28.0 ± 0.01 ab	27.3 ± 0.35 ab
**TVP + Psyllium**	2.0 ± 0.03 a	1.20 ± 0.01 a	1.9 ± 0.00 a	27.3 ± 0.35 a	27.0 ± 0.00 a
**TVP + mix**	2.2 ± 0.08 ab	1.14 ± 0.10 a	2.0 ± 0.03 a	29.5 ± 0.71 b	28.0 ± 0.00 b

Values are expressed as means ± standard deviation of three independently prepared mixtures (n = 3). Different letters within the same column indicate significant differences (Tukey-HSD, *p* ≤ 0.05). WAC: water absorption capacity; WSI: water solubility index; OAC: oil absorption capacity; FC: foaming capacity; FS: foaming stability.

## Data Availability

Data are contained within the article or Supplementary Materials.

## Supplementary Materials

The following supporting information can be downloaded at: https://www.mdpi.com/article/10.3390/foods15213785/s1, Figure S1: Mixolab profile of pea-based texturized vegetable protein (TVP) systems without hydrocolloids or supplemented with 1% (w/w) methylcellulose (MC), hydroxypropyl methylcellulose (HPMC), psyllium, or their ternary mixture (mix; MC:HPMC:psyllium = 1:1:1). A representative curve for each sample is reported; Table S1: Moisture content and water activity (aw) of uncooked and cooked pea-based texturized vegetable protein (TVP) burgers prepared without hydrocolloids or with 1% (w/w) methylcellulose (MC), hydroxypropyl methylcellulose (HPMC), psyllium, or their ternary mixture (mix; MC:HPMC:psyllium = 1:1:1), after mixing at 5, 10, 15, and 25 °C. Values are expressed as mean ± standard deviation of two independent bulk preparations (n = 2); two burger subsamples were analysed per preparation.

## Abbreviations

The following abbreviations are used in this manuscript:

BU	Brabender units
FC	Foaming capacity
FS	Foaming stability
HPMC	Hydroxypropyl methylcellulose
MC	Methylcellulose
OAC	Oil absorption capacity
PCA	Principal component analysis
TVP	Texturized vegetable protein
WAC	Water absorption capacity
WSI	Water solubility index
