# Supplementary material for: Effects of Hydrocolloid Type and Mixing Temperature on the Properties of Pea-Based Texturized Vegetable Protein Burgers
Source: Foods. 2026 Oct 22;15(21):3785. doi: 10.3390/foods15213785 (PMC13763351; doi:10.3390/foods15213785)
Supplement: Supplementary file 1 [file foods-15-03785-s001.zip › foods-4567110-supplementary.pdf]

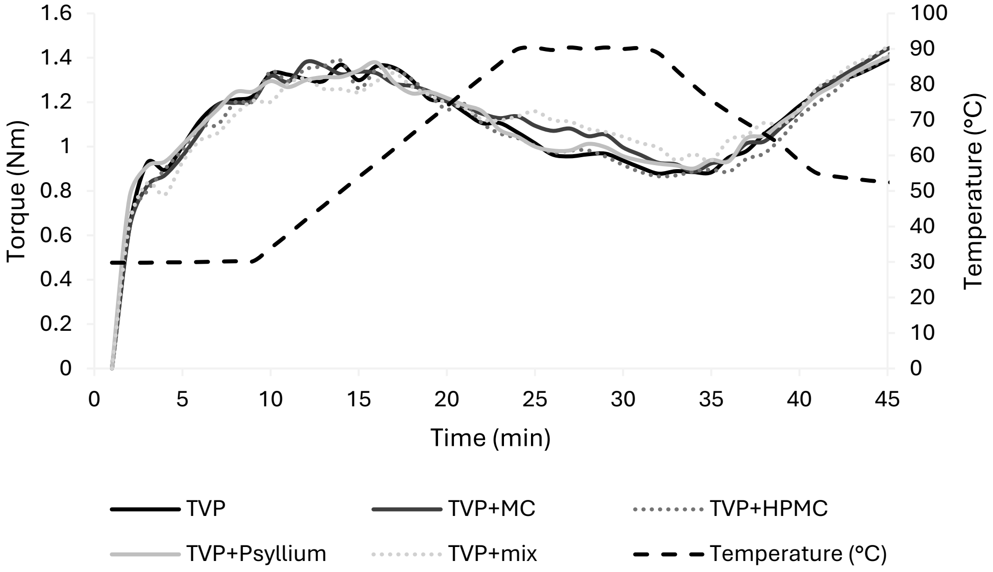

Figure S1. Mixolab profile of pea-based texturized vegetable protein (TVP) systems without hydrocolloids or supplemented with 1% (w/w) methylcellulose (MC), hydroxypropyl methylcellulose (HPMC), psyllium, or their ternary mixture (mix; MC:HPMC:psyllium = 1:1:1). A representative curve for each sample is reported.

Table S1. Moisture content and water activity (aw) of uncooked and cooked pea-based texturized vegetable protein (TVP) burgers prepared without hydrocolloids or with 1% (w/w) methylcellulose (MC), hydroxypropyl methylcellulose (HPMC), psyllium, or their ternary mixture (mix; MC:HPMC:psyllium = 1:1:1), after mixing at 5, 10, 15, and 25 °C. Values are expressed as mean ± standard deviation of two independent bulk preparations (n = 2); two burger subsamples were analysed per preparation.

**Mixing temperature**	**Burger**	**Parameter**	**TVP**	**+ MC**	**+ Psyllium**	**+ HPMC**	**+ MIX**
**5 °C**	Raw	Moisture (%)	68.85 ± 0.08	68.22 ± 0.30	68.83 ± 0.56	68.47 ± 0.07	68.37 ± 0.21
		aw	0.994 ± 0.001	0.993 ± 0.000	0.993 ± 0.000	0.992 ± 0.001	0.993 ± 0.001
	Cooked	Moisture (%)	55.35 ± 0.09	56.54 ± 0.48	55.78 ± 0.27	56.07 ± 0.36	55.20 ± 0.51
		aw	0.992 ± 0.001	0.991 ± 0.000	0.992 ± 0.001	0.993 ± 0.000	0.992 ± 0.000
**10 °C**	Raw	Moisture (%)	68.50 ± 0.32	68.63 ± 0.45	69.59 ± 0.48	68.32 ± 0.31	68.99 ± 0.95
		aw	0.996 ± 0.000	0.992 ± 0.001	0.993 ± 0.001	1.000 ± 0.001	1.000 ± 0.001
	Cooked	Moisture (%)	57.31 ± 0.26	59.25 ± 0.15	59.82 ± 0.10	59.05 ± 1.02	58.61 ± 0.32
		aw	0.992 ± 0.001	0.985 ± 0.004	0.993 ± 0.000	0.991 ± 0.001	0.991 ± 0.000
**15 °C**	Raw	Moisture (%)	68.60 ± 0.24	68.36 ± 0.24	69.25 ± 0.22	69.00 ± 0.80	68.44 ± 0.25
		aw	0.998 ± 0.001	0.998 ± 0.000	0.999 ± 0.000	1.000 ± 0.001	1.000 ± 0.001
	Cooked	Moisture (%)	58.93 ± 0.27	59.72 ± 0.03	58.76 ± 0.31	58.34 ± 0.27	58.26 ± 0.13
		aw	0.993 ± 0.001	0.997 ± 0.001	0.989 ± 0.001	0.992 ± 0.000	0.992 ± 0.000
**25 °C**	Raw	Moisture (%)	68.99 ± 0.62	69.06 ± 0.26	69.26 ± 0.38	68.40 ± 0.39	69.28 ± 0.52
		aw	0.990 ± 0.001	0.995 ± 0.000	0.990 ± 0.000	0.990 ± 0.001	0.988 ± 0.001
	Cooked	Moisture (%)	57.58 ± 0.60	59.20 ± 0.46	58.66 ± 0.16	59.84 ± 0.58	58.40 ± 0.45
		aw	0.994 ± 0.001	0.993 ± 0.000	0.993 ± 0.000	0.992 ± 0.001	0.993 ± 0.001
